# Combined Treatment with Herbal Medicine and Drug Ameliorates Inflammation and Metabolic Abnormalities in the Liver of an Amyotrophic Lateral Sclerosis Mouse Model

**DOI:** 10.3390/antiox11010173

**Published:** 2022-01-17

**Authors:** Hee Ra Park, Eun Jin Yang

**Affiliations:** KM Medicine Science Research Division, Korea Institute of Oriental Medicine (KIOM), Daejeon 34054, Korea; hrpark0109@kiom.re.kr

**Keywords:** amyotrophic lateral sclerosis, Bojungikgi-tang, riluzole, liver function, inflammation, cellular stress, oxidative phosphorylation

## Abstract

To date, no effective drugs exist for amyotrophic lateral sclerosis (ALS), although riluzole (RZ) and edaravone have been approved for treatment. We previously reported that Bojungikgi-tang (BJIGT) improved motor activity through anti-inflammatory effects in the muscle and spinal cord of hSOD1^G93A^ mice. Therefore, whether combined treatment with BJIGT and RZ synergistically affects liver function in hSOD1^G93A^ mice was investigated. Two-month-old male hSOD1^G93A^ mice were treated with BJIGT (1 mg/g) and RZ (8 μg/g) administered orally for 5 weeks. Drug metabolism and liver function tests of serum and liver homogenates were conducted. mRNA expression levels of cytochrome P450 (CYP) isozymes, inflammatory cytokines, metabolic factors, and mitochondrial oxidative phosphorylation (OXPHOS) subunits were examined using qPCR and Western blotting. Combined administration of BJIGT and RZ did not alter mRNA expression levels of drug-metabolism-related isozymes (CYP1A2 and CYP3A4) but significantly decreased the activity of liver-function-related enzymes (AST, ALT, ALP, and LDH). Increased expression of inflammatory cytokines (IL-1β, TNF-α, and IL-6) and of intracellular stress-related proteins (Bax, AMPKα, JNK, and p38) was reduced by the combined treatment in hSOD1^G93A^ mice compared to that in control mice. Combined administration reduced the mRNA expression of metabolism-related factors and the expression of OXPHOS subunits. Elevated ATP levels and mitochondrial-fusion-associated protein were decreased after co-administration. Co-administration of BJIGT and RZ did not cause liver damage or toxicity but rather restored liver function in hSOD1^G93A^ mice. This suggests that this combination can be considered a candidate therapeutic agent for ALS.

## 1. Introduction

Amyotrophic lateral sclerosis (ALS) is a neurodegenerative disease caused by progressive motor neuron death and is classified as a neuromuscular disease owing to muscle atrophy leading to respiratory failure and death. Familial ALS is caused by genetic factors such as SOD1A, C9orf72, TARDBP, and FUS mutations, but the majority of cases are sporadic ALS. Neurodegenerative diseases are often characterized by multiple pathological mechanisms, and those underlying ALS include protein misfolding, inflammation, oxidative stress, and mitochondrial dysfunction [1]. Patients with ALS and transgenic mice both have metabolic abnormalities and energy imbalance, including glucose intolerance and dyslipidemia [2,3]. These abnormalities are related to disease progression and susceptibility, although the relationship between metabolic defects and ALS pathogenesis is unclear [4].

The motor neurons and glia of patients with ALS show that mitochondrial and glycolytic energy metabolic pathways are impaired [5]. Mitochondrial dysfunction occurs in hSOD1^G93A^ mice, the animal model of ALS, including mitochondrial degeneration, vacuolization, and oxidative phosphorylation (OXPHOS) impairment in motor neurons [6,7]. Furthermore, Scaricamazza et al. reported that metabolic dysfunctions, such as hypermetabolism and ATP synthesis decline, are attributed to mitochondrial dysfunction in the skeletal muscle of hSOD1^G93A^ mice [8]. Recent studies have shown that mitochondrial abnormalities are closely related to ALS, with impaired mitochondrial OXPHOS likely contributing to ALS pathology [9,10].

The liver is the main organ involved in the systemic homeostasis of lipometabolism and glycometabolism. Liver abnormalities such as inactive micronodular cirrhosis and elevation of natural killer T (NKT) cells have been detected in patients and animal models of ALS [11]. Nakano et al. showed liver dysfunction in patients with ALS [12]. In our previous study, we demonstrated that the levels of inflammation and oxidative-stress-related proteins are significantly increased with disease progression in hSOD1^G93A^ mice [13].

Herbal medicines target multiple biological pathways and are composed of complex mixtures compared to Western drugs [14]. Stickel et al. reported that the anti-inflammatory and anti-oxidative properties of herbal medicine are beneficial in chronic liver disease, although it is controversial whether herbal medicines in combination are toxic [15]. Bojungikgi-tang (BJIGT; Buzhongyiqi-tang in Chinese, Hochuekki-to in Japanese) is a polyherbal medicine used for anti-inflammation and anti-oxidation in cancer and neurological diseases [16,17,18]. In a previous study, we demonstrated that BJIGT improves motor function and exerts anti-inflammatory and anti-oxidative effects in the spinal cord and skeletal muscle of hSOD1^G93A^ mice [16]. In addition, previous studies demonstrated that combined treatment with BJIGT and cisplatin is synergistically effective in apoptosis and autophagy in vitro [19]. Riluzole (RZ), an FDA-approved drug, has been used to treat ALS but prolongs survival by only 3–4 months [20]. Therefore, we hypothesized that combined treatment with BJIGT and RZ would safely improve liver function in hSOD1^G93A^ mice.

## 2. Materials and Methods

### 2.1. Animals

Hemizygous transgenic B6SJL male mice (5 weeks old) carrying a glycine-to-alanine mutation at the 93rd codon of the cytosolic Cu/Zn superoxide dismutase gene (hSOD1^G93A^) were purchased from the Jackson Laboratory (Bar Harbor, ME, USA) and maintained under temperature- and light-controlled conditions (20–23 °C, 12 h light/12 h dark cycle) with food and water provided ad libitum. All animals were acclimatized for 7 days prior to drug administration. The Institutional Animal Care Committee of the Korea Institute of Oriental Medicine approved the experimental protocol, and all experiments were performed in accordance with the guidelines of the U.S. National Institutes of Health guidelines and the Animal Care and Use Committee at KIOM.

### 2.2. Drug Administration

**Figure 1:**
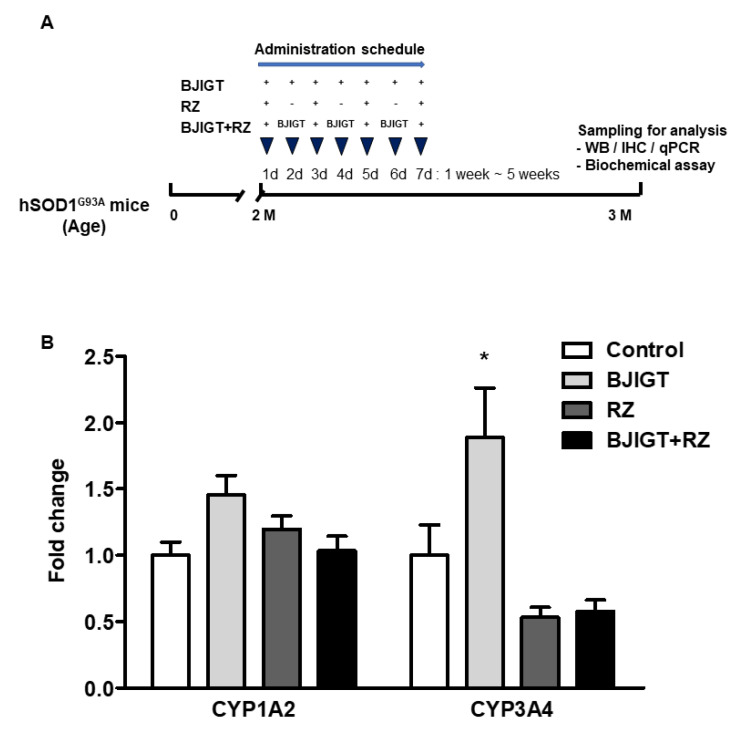
Experimental scheme for the in vivo study and relative expression level of CYP enzymes in the liver of hSOD1^G93A^ mice. (**A**) In vivo experimental design. Two-month-old hSOD1^G93A^ mice were administered autoclaved distilled water (control, *n* = 5 mice), BJIGT (1 mg/g, *n* = 5 mice), RZ (8 μg/g, *n* = 7 mice), or a combination of BJIGT and RZ (*n* = 9 mice) for 5 weeks. Mice were sacrificed for analysis 5 weeks after drug administration. (**B**) The expression levels of CYP1A2 and CYP3A4 mRNAs in each group. * *p* < 0.05 vs. Control. *n* = 5–9 mice per experimental group.

BJIGT and RZ were purchased from Hankookshinyak (Nonsan, Chungnam, Korea) and Calbiochem (Darmstadt, Germany), respectively. BJIGT was dissolved in autoclaved distilled water, and RZ was dissolved in 1% DMSO. Control groups were administered an equal volume of autoclaved distilled water and 1% DMSO. Mice were randomly divided into four groups (Figure 1A): (1) hSOD1^G93A^ transgenic mice (control, *n* = 5 mice); (2) hSOD1^G93A^ mice with BJIGT administration (BJIGT, *n* = 5 mice); (3) hSOD1^G93A^ mice with RZ administration (RZ, *n* = 7 mice); (4) hSOD1^G93A^ mice with BJIGT and RZ administration (BJIGT + RZ, *n* = 9 mice). BJIGT (1 mg/g) was administered orally once per day for 5 weeks in the 2-month-old mice in BJIGT and BJIGT + RZ groups. RZ (8 μg/g) was administered orally every other day for 5 weeks to the 2-month-old mice in BJIGT and RZ groups. The dose was based on previous results for adult human subjects (5 g/60 kg body weight/day) [21].

### 2.3. Tissue Preparation

For biochemical analyses, serum was collected by centrifugation at 2000× *g* for 15 min at 4 °C, and livers were homogenized in the assay buffer and centrifuged (12,000 rpm for 15 min at 4 °C). For Western blotting, livers (50 mg) were homogenized in RIPA buffer (Biosesang, Seongnam, Korea), protease inhibitor cocktail, and phosphatase inhibitor cocktail (Thermo Fisher Scientific, Waltham, MA, USA) and centrifuged (12,000 rpm for 15 min at 4 °C). All serum and tissue supernatants were stored at −80 °C until required for the assay. For histological analyses, mice were anesthetized with 20 mg/mL avertin and perfused intracardially with 50 mL of 0.9% saline, followed by 50 mL of 4% paraformaldehyde in PBS. After perfusion, the liver was postfixed for 24 h in fixative at 4 °C and embedded in paraffin. Paraffin-embedded liver tissues were sectioned into 5-μm-thick sections using a microtome and mounted on slides.

### 2.4. Western Blot Analysis

Liver homogenates were solubilized in SDS-polyacrylamide gel electrophoresis sample buffer, and protein concentrations were determined using a BCA assay kit (Thermo Fisher Scientific) with a bovine serum albumin standard. Samples (20 μg protein per lane) were separated on SDS-polyacrylamide gels and transferred by electrophoresis to polyvinylidene difluoride transfer membranes (Bio-Rad, Hercules, CA, USA). Membranes were then incubated with specific antibodies. Anti-IL-1β (ab9722), TNF-α (ab1793), mitofusin (MFN) 1/2 (ab57602), optic atrophy 1 (OPA1) (ab157457), and α-tubulin (ab4074) were obtained from Abcam. Antibodies against Bax (sc-493), Bcl-2 (sc-7382), and transferrin (sc-22597) were purchased from Santa Cruz Biotechnology (Dallas, TX, USA). Anti-phosphorylated (p) JNK (#9251), p-p38 (#9215), total (t) JNK (#9252), t-p38 (#9212), p-AMPKα (#2531). t-AMPKα (#5831) and COX IV (#4850) were purchased from Cell Signaling Technology (Danvers, MA, USA). Proteins were detected using a Bio-Rad Clarity Western ECL substrate and a ChemiDoc Touch Imaging System. The relative band intensities were calculated using Image Lab software version 6.1.0 (Bio-Rad). Pre-stained blue markers were used to determine molecular weights.

### 2.5. Quantitative Reverse Transcription PCR

Total RNA was extracted from 30 mg of each liver sample (easy-spin^TM^ total RNA extraction kit, iNtRON Biotechnology, Seongnam, Korea) and quantified (NanoDrop spectrophotometer, Thermo Fisher Scientific). One microgram of RNA sample was reverse-transcribed to cDNA, and quantitative PCR with SYBR green was performed (Bio-Rad) and an Applied Biosystems QuantStudio^TM^ 6 flex (Thermo Fisher Scientific) was used for analyses. The housekeeping gene GAPDH was used as an internal control. The primer sets are shown in Supplementary Table S1.

### 2.6. Hematoxylin and Eosin (H&E) Staining

After deparaffinization, sections were stained with H&E. Sections were stained with hematoxylin solution for 5 min, washed in running tap water, and stained with eosin for 30 s. After washing, sections were dehydrated and mounted on slides. Tissues were randomly selected from each group, and blinded analysis of H&E staining was performed. Images were acquired using an Olympus BX53 microscope (Olympus, Tokyo, Japan).

### 2.7. Statistical Analysis

Data analyses were evaluated by one-way ANOVA followed by Dunnett’s test using GraphPad PRISM software version 5.02 (GraphPad Software Inc., San Diego, CA, USA). Results are expressed as the mean ± standard error of the mean (SEM), and *p*-values < 0.05 were considered significant.

## 3. Results

### 3.1. Combination Treatment Did Not Affect Gene Expression Levels of Cytochrome P450 Enzymes in the Liver of hSOD1^G93A^ Mice

To determine whether combined administration of BJIGT and RZ caused toxicity in the livers of hSOD1^G93A^ mice during drug metabolism, we evaluated mRNA expression levels of CYP1A2 and CYP3A4. Combined administration of BJIGT and RZ did not affect the hepatic CYP1A2 and CYP3A4 levels, which changed by 1.0-fold and 0.6-fold, respectively (Figure 1B). The expression level of the hepatic CYP1A2 gene was not significant in the BJIGT or RZ groups, either alone or in combination (Figure 1B). BJIGT administration alone significantly increased the expression levels of CYP3A4 in the liver by 1.9-fold (Figure 1B). RZ administration alone and in combination with BJIGT demonstrated no effect on CYP3A4 mRNA levels. This result suggests that combined administration of BJIGT and RZ did not significantly interfere with hepatic CYP enzymes and that this novel combination does not induce harmful effects during drug metabolism in the liver.

### 3.2. Combined Administration of BJIGT and RZ Attenuated Abnormal Liver Function in hSOD1^G93A^ Mice

**Figure 2:**
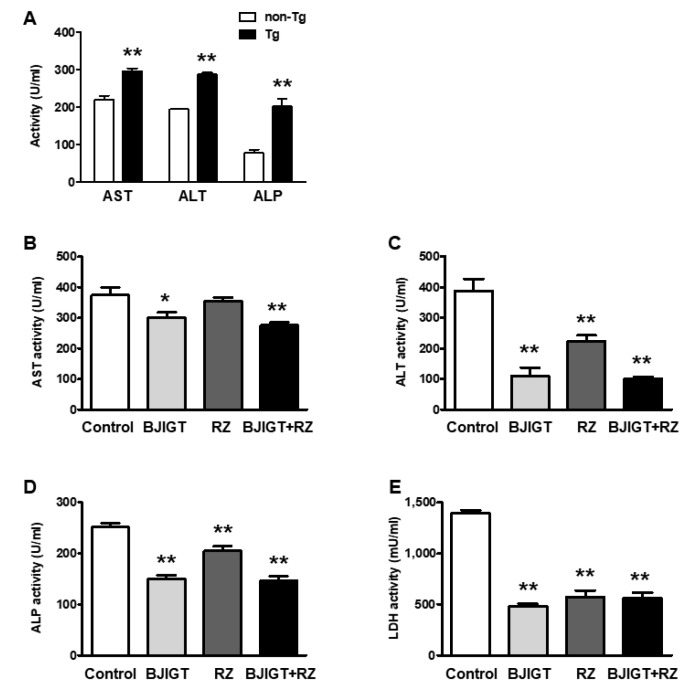
Effects of BJIGT and RZ combination treatment on serum AST, ALT, ALP, and LDH activity of hSOD1^G93A^ mice. (**A**) Serum levels of AST, ALT, ALP, and LDH in non-transgenic mice (non-Tg) and hSOD1^G93A^ mice (Tg) (*n* = 4 mice/group). (**B**–**E**) Serum levels of AST (**B**), ALT (**C**), ALP (**D**), and LDH (**E**) in each group. Data are presented as the mean ± SEM; * *p* < 0.05, ** *p* < 0.01, vs. control. *n* = 5–9 mice per experimental group.

As drug combinations may induce liver toxicity, we performed liver function tests in the serum and livers of hSOD1^G93A^ mice administered BJIGT and RZ. We first checked the AST, ALT, and ALP activity in the serum of non-transgenic (non-Tg) mice and hSOD1^G93A^ mice. In the serum of hSOD1^G93A^ mice, the activity of AST, ALT, and ALP were significantly higher than those in that of non-Tg mice, by 1.4-fold, 2.6-fold, and 1.5-fold, respectively (Figure 2A), suggesting that hSOD1^G93A^ mice experienced liver dysfunctions.

Compared with that in control mice, only BJIGT administration significantly reduced the activity of AST, ALT, and ALP in the serum of hSOD1^G93A^ mice, by 0.8-fold, 0.3-fold, and 0.6-fold, respectively (Figure 2B–D). In the serum of mice treated with RZ alone, ALT and ALP activity, but not AST activity, was decreased compared with that in control mice, by 0.6-fold and 0.8-fold, respectively (Figure 2B–D). Combined administration of BJIGT and RZ significantly reduced the activity of AST, ALT, and ALP by 0.7-fold, 0.3-fold, and 0.6-fold, respectively, compared with that of control mice (Figure 2B–D). BJIGT and RZ administered alone and in combination decreased LDH activity in the serum by 0.4-fold compared with that in control mice (Figure 2E).

**Figure 3:**
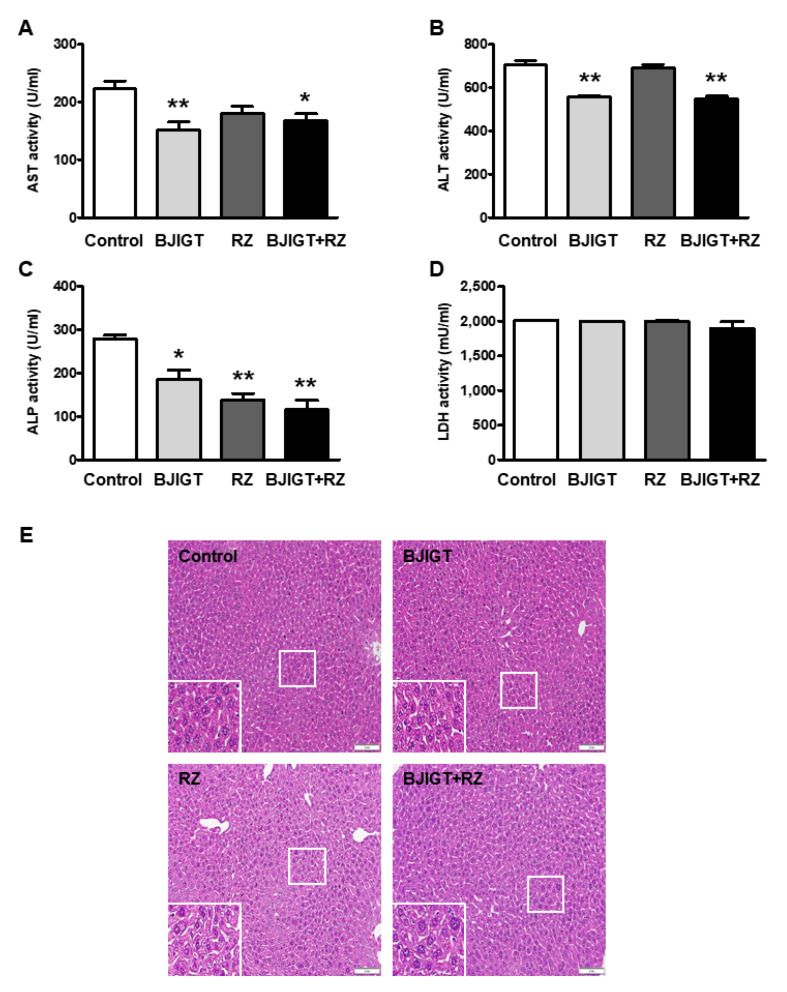
Effects of BJIGT and RZ combination treatment on hepatic function in hSOD1^G93A^ mice. (**A**–**D**) Levels of AST (**A**), ALT (**B**), ALP (**C**), and LDH (**D**) in the liver of each group. Data are presented as the mean ± SEM; * *p* < 0.05, ** *p* < 0.01, vs. control. *n* = 5–9 mice per experimental group (**E**). Representative images of H&E staining of the liver histological presentations of each group. The inserted boxes show magnified images. Scale bar = 2 mm.

In the liver, AST, ALT, and ALP activities were also decreased in BJIGT-treated mice by 0.7-fold, 0.8-fold, and 0.7-fold, respectively, compared with those in control mice (Figure 3A–C). In RZ-treated mice, hepatic ALP activity was reduced compared with that in control mice, by 0.5-fold (Figure 3A–C). These results indicated that abnormally elevated levels of enzymes that indicate liver function were ameliorated by BJIGT or RZ administration. In the livers of hSOD1^G93A^ mice in the BJIGT and RZ combination group, activities of AST, ALT, and ALP were significantly decreased by 0.8-fold, 0.8-fold, and 0.4-fold, respectively, compared with that in those of control mice (Figure 3A–C). However, LDH levels in livers of mice treated with BJIGT or RZ alone or in combination were not affected (Figure 3D).

H&E staining was performed to determine whether the combined administration of BJIGT and RZ affected histological liver pathologies, such as cell death, fibrosis, or cirrhosis. Liver sections from mice subjected to combined or separate administration of BJIGT and RZ did not exhibit any pathological patterns compared with those from control mice (Figure 3E). These results suggested that increased liver-function-related enzymes were significantly alleviated by the combined administration of BJIGT and RZ without any drug toxicity or liver injury.

### 3.3. Increased Inflammatory Cytokines in the Liver of hSOD1^G93A^ Mice Were Ameliorated by Combined Administration of BJIGT and RZ

**Figure 4:**
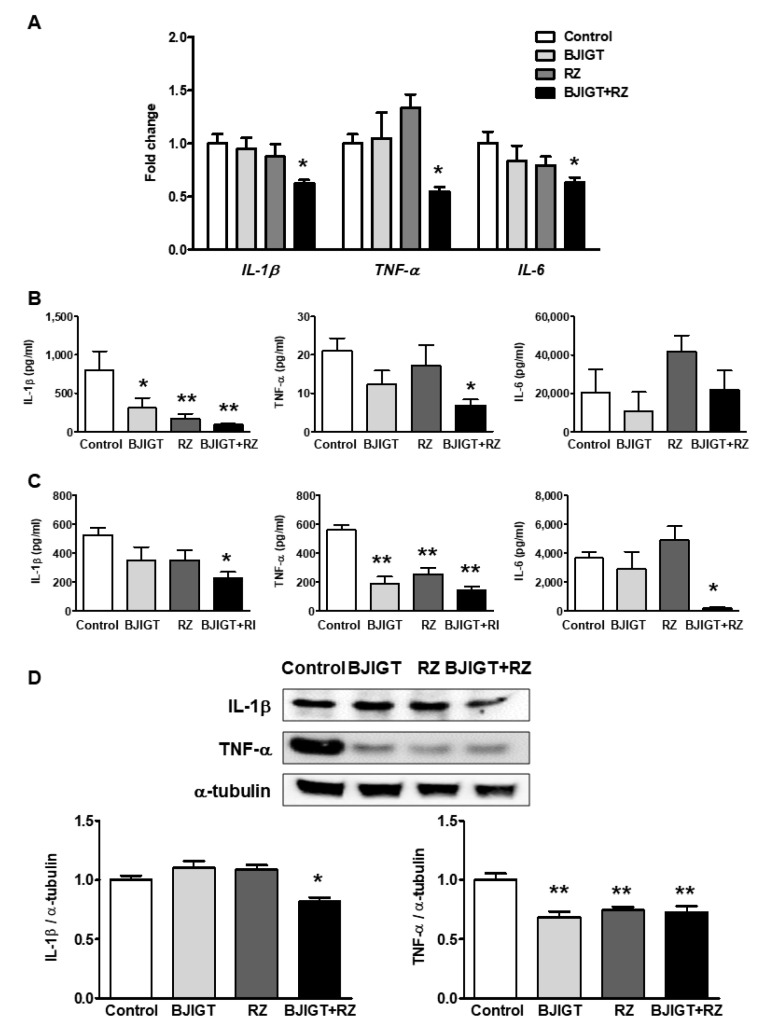
Combined administration of BJIGT and RZ reduced inflammatory cytokines in the serum and liver of hSOD1^G93A^ mice. (**A**) Gene expression of *IL-1β*, *TNF-α*, and *IL-6* in the liver. mRNA was quantified by qPCR, and the resulting values were expressed as relative mRNA expression levels after normalization to GAPDH. (**B**,**C**) The concentration of inflammatory cytokines (IL-1β, TNF-α, IL-6) in serum (**B**) and liver (**C**) was determined by a flow cytometry bead-based assay. (**D**) Representative bands of IL-1β, TNF-α, and α-tubulin expression. Densitometric analysis indicates the quantification of IL-1β, TNF-α, and transferrin expressed as relative expression levels after normalization to α-tubulin. Data are presented as the mean ± SEM; * *p* < 0.05, ** *p* < 0.01, vs. control. *n* = 5–9 mice per experimental group.

To determine the effect of the BJIGT and RZ combination on liver inflammation, we examined mRNA expression of inflammatory cytokines in the livers of hSOD1^G93A^ mice treated with BJIGT and RZ individually or in combination. Interestingly, the combined administration of BJIGT and RZ significantly reduced mRNA expression levels of hepatic inflammatory cytokines, especially *IL-1β*, *TNF-α*, and *IL-6*, compared with those in control mice, by 0.6-fold, 0.6-fold, and 0.6-fold, respectively (Figure 4A). BJIGT or RZ administered separately did not cause changes in mRNA expression levels of these genes (Figure 4A).

To confirm protein expression levels of inflammatory cytokines in the serum and liver, we performed flow cytometry using a mouse inflammation multiplex assay. The combined administration of BJIGT and RZ significantly reduced the levels of IL-1β and TNF-α by 0.1-fold and 0.4-fold, respectively, compared with those in control mice (Figure 4B). Administration of BJIGT or RZ alone significantly reduced the levels of IL-1β in serum by 0.4-fold or 0.2-fold, respectively, compared with levels in control mice (Figure 4B). In the liver homogenates, combined administration of BJIGT and RZ reduced the levels of IL-1β (by 0.4-fold), TNF-α (by 0.3-fold), and IL-6 (by 0.1-fold) compared to those in the control group (Figure 4C). Levels of TNF-α in the liver also decreased with BJIGT or RZ alone compared to those in control mice by 0.3-fold and 0.4-fold, respectively (Figure 4C). Western blotting revealed that the combined administration of BJIGT and RZ also decreased the protein expression of IL-1β and TNF-α in the liver by 0.8-fold and 0.7-fold, respectively, compared with that in control mice (Figure 4D). BJIGT and RZ administered separately resulted in a reduction in TNF-α expression but not IL-1β in the liver of hSOD1^G93A^ mice.

### 3.4. Cellular Stress-Mediated Proteins Were Attenuated by Combined Administration of BJIGT and RZ in the Liver of hSOD1^G93A^ Mice

**Figure 5:**
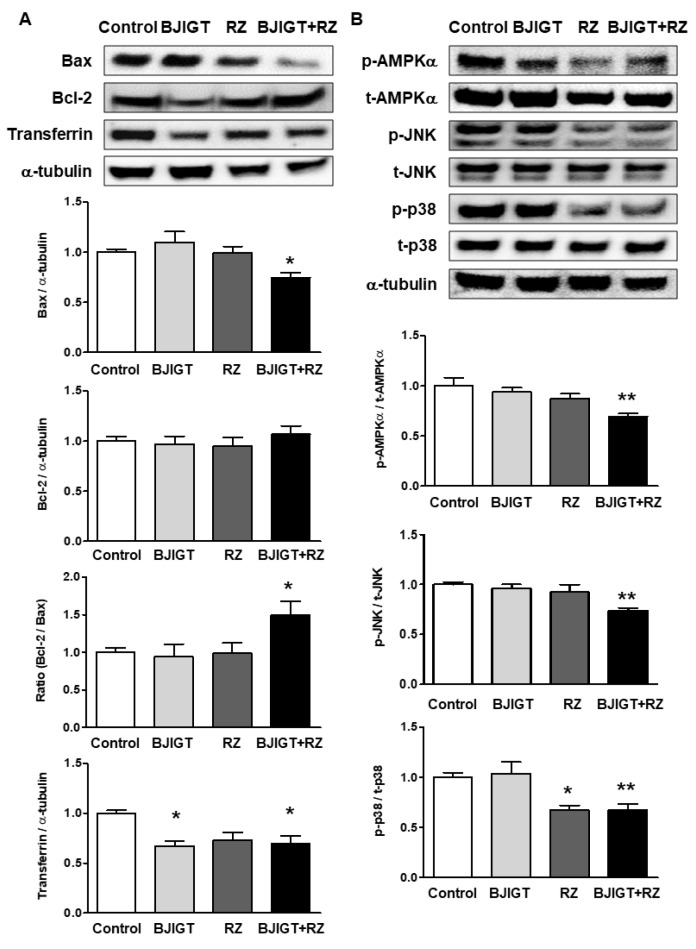
Cellular stress-related factors were decreased by BJIGT and RZ administration in the liver of hSOD1^G93A^ mice. (**A**) Representative bands of Bax, Bcl-2, transferrin, and α-tubulin expression are shown. Densitometric analysis indicated the quantification of Bax, Bcl-2, and transferrin expressed as relative expression levels after normalization to α-tubulin. (**B**) Representative bands of p-AMPKα, t-AMPKα, p-JNK, t-JNK, p-p38, t-p38, and α-tubulin expression are shown. Densitometric analysis indicated the quantification of p-AMPKα, p-JNK, and p-p38, expressed as the ratio of p-AMPK/t-AMPK α, p-JNK/t-JNK, or p-p38/t-p38. Data are presented as the mean ± SEM; * *p* < 0.05, ** *p* < 0.01, vs. control. *n* = 5–9 mice per experimental group.

Combined administration of BJIGT and RZ resulted in a lower expression level of Bax (by 0.7-fold) in relation to that of Bcl-2 and a higher Bax/Bcl-2 ratio (by 1.5-fold) compared to that in control mice (Figure 5A). The protein expression level of transferrin was reduced in mice administered a combination of BJIGT and RZ, as well as in mice administered BJIGT alone, by 0.7-fold (Figure 5A). As cellular stress induces the activation of intracellular protein kinases in the liver, we examined the expression of p-AMPK, p-JNK, and p-p38 in the liver. The combined administration of BJIGT and RZ had a much better reductive effect than either one administered alone, by 0.7-fold, compared with control mice (Figure 5B). Mice treated with RZ alone also showed a lower expression level of p-p38 in the liver by 0.7-fold (Figure 5B). Increased cellular stress-related protein expression in the liver of hSOD1^G93A^ mice was reduced by the combined administration of BJIGT and RZ.

### 3.5. Metabolic Dysfunction in the Liver of hSOD1^G93A^ Mice Was Improved by Combined Administration of BJIGT and RZ

**Figure 6:**
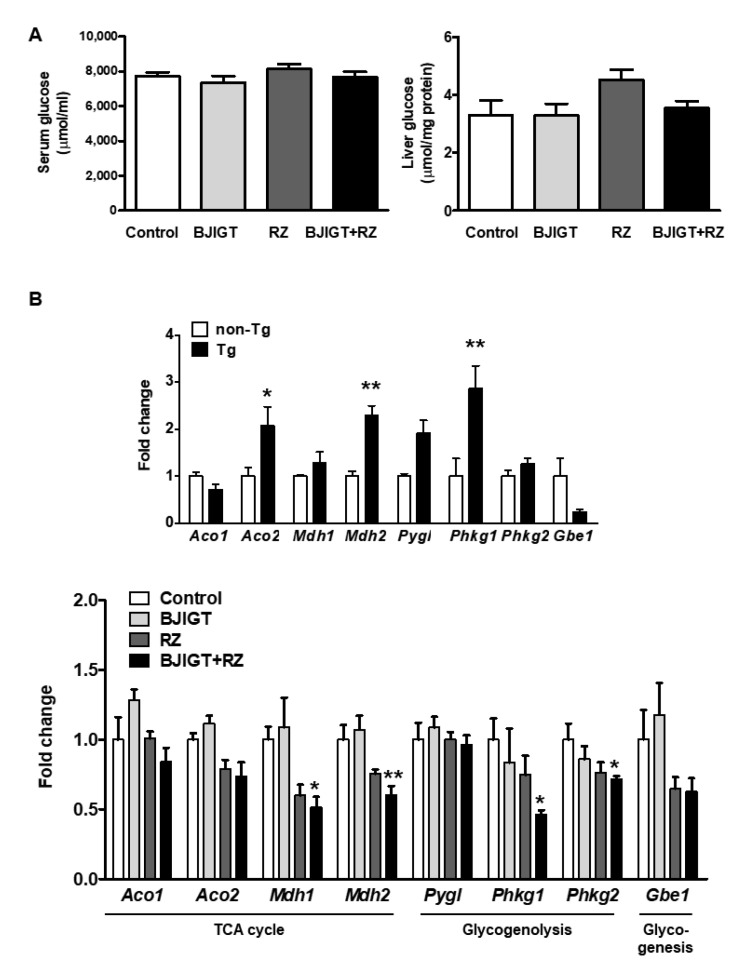
Combined administration of BJIGT and RZ ameliorated metabolism-related protein expression in the liver of hSOD1^G93A^ mice. (**A**) Levels of glucose in the serum and liver of each group. (**B**) Gene expression of the TCA cycle (*Aco1*, *Aco2*, *Mdh1*, *Mdh2*), glycogenolysis (*Pygl*, *Phkg1*, *Phkg2*), and glycogenesis (*Gbe1*) in the livers of non-Tg and Tg mice. * *p* < 0.05, ** *p* < 0.01 vs. non-Tg. *n* = 4 mice per group. Gene expression of the TCA cycle (*Aco1*, *Aco2*, *Mdh1*, *Mdh2*), glycogenolysis (*Pygl*, *Phkg1*, *Phkg2*), and glycogenesis (*Gbe1*) in the liver. mRNA was quantified by qPCR, and the resulting values were expressed as relative mRNA expression levels after normalization to GAPDH. Data are presented as the mean ± SEM; * *p* < 0.05, ** *p* < 0.01, vs. control. *n* = 5–9 mice per experimental group.

We examined the effect of the BJIGT and RZ combination on glucose levels in the serum and liver of hSOD1^G93A^ mice. However, in this group, glucose concentrations in the serum and liver were not altered compared to those in control mice (Figure 6A).

We also evaluated the mRNA expression of eight genes associated with metabolic-pathway-related factors to determine whether alterations occurred in certain metabolic pathways. In the TCA cycle, we examined mRNA expression levels of the isoform of aconitase (cytosolic *Aco1* and mitochondrial *Aco2*) and malate dehydrogenase (cytosolic *Mdh1* and mitochondrial *Mdh2*). In the glycogenolysis pathway, we examined glycogen phosphorylase (*Pygl*) and phosphorylase kinase (*Phkg1* and *Phkg2*). We also examined the expression level of glycogen branching enzyme (*Gbe1*), which is involved in glycogen synthesis.

The expression levels of several factors, especially *Aco2*, *Mdh2*, and *Phkg1*, were significantly increased in hSOD1^G93A^ Tg mice compared with those in non-Tg mice, by 2.1-fold, 2.3-fold, and 2.0-fold, respectively (Figure 6B), indicating that metabolism in the liver of hSOD1^G93A^ mice was dramatically altered, which is consistent with recent evidence. Next, we evaluated whether metabolism pathway changes were modulated by the combination of BJIGT and RZ. In the TCA cycle, mRNA expression levels of *Mdh1* and *Mdh2* were reduced in hSOD1^G93A^ mice administered combined BJIGT and RZ by 0.5-fold and 0.6-fold, respectively, compared with those in control mice (Figure 6B). In the glycogenolysis pathway, mRNA expression levels of *Phkg1* and *Phkg2* were decreased in the livers of mice administered combined BJIGT and RZ compared with those in control mice, by 0.5-fold and 0.7-fold, respectively (Figure 6B).

### 3.6. Dysfunction of the OXPHOS System and Abnormal Mitochondrial Fusion in the Liver of hSOD1^G93A^ Mice Was Improved by Combined Administration of BJIGT and RZ

**Figure 7:**
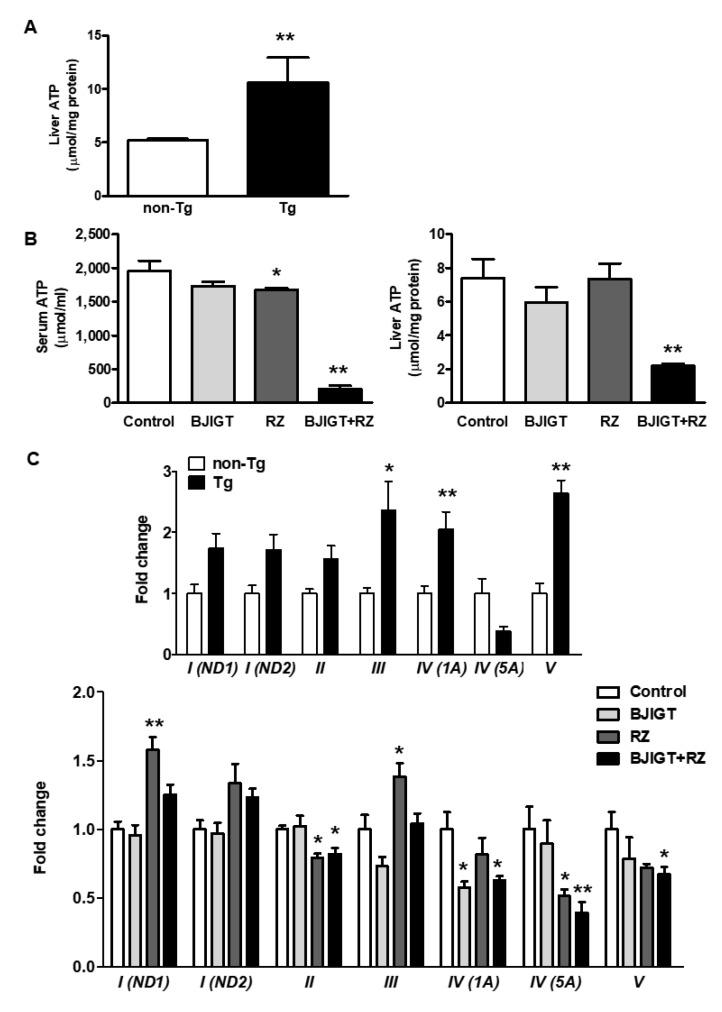

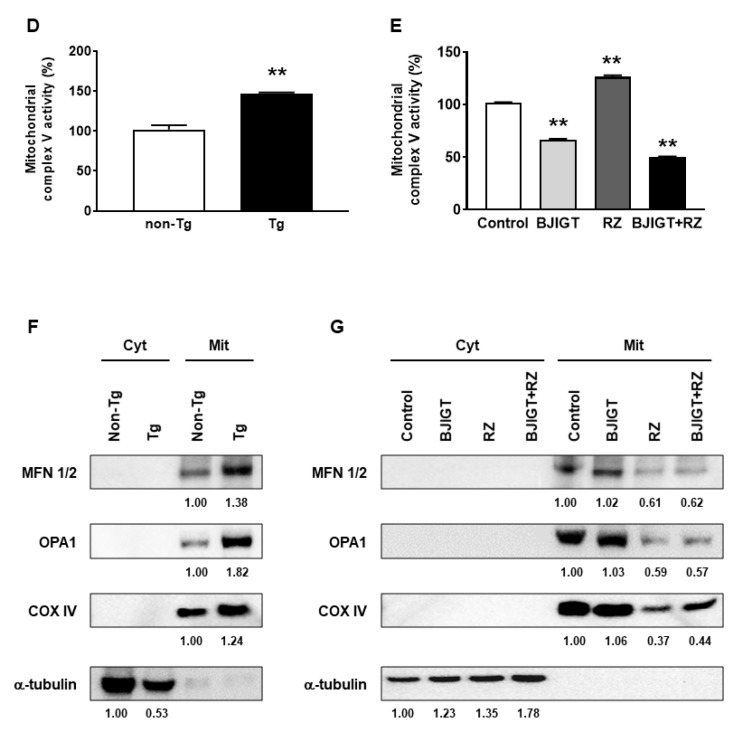
Combined administration of BJIGT and RZ reduced protein expression of mitochondrial fusion markers and mitochondrial OXPHOS mRNA expression in the liver of hSOD1^G93A^ mice. (**A**) Levels of ATP in the livers of non-Tg and Tg mice. ** *p* < 0.01 vs. non-Tg. *N* = 3 mice per group. (**B**) ATP levels in the serum and liver of each group. (**C**) Gene expression of complex I (ND1 and ND2), complex II, complex III, complex IV (1A and 5A), and complex V in the livers of non-Tg and Tg mice. * *p* < 0.05, ** *p* < 0.01 vs. non-Tg. *N* = 4 mice per group. Expression of complex I (ND1 and ND2), complex II, complex III, complex IV (cox1a and cox5a), and complex V in the liver. mRNA was quantified by qPCR, and the resulting values were expressed as relative mRNA expression levels after normalization to GAPDH. Data are presented as the mean ± SEM; * *p* < 0.05, ** *p* < 0.01, vs. control. *N* = 5–9 mice per experimental group. (**D**) The activity of complex V in the livers of non-Tg and Tg mice. ** *p* < 0.01 vs. non-Tg. *N* = 3 mice per group. (**E**) The activity of complex V in the livers of each group. Data are presented as the mean ± SEM; * *p* < 0.05, ** *p* < 0.01, vs. control. *n* = 5–9 mice per experimental group. (**F**) The cytosol and mitochondrial proteins were obtained from pooled liver tissue from non-Tg or Tg mice. Representative bands of MFN 1/2, OPA1, COX IV, and α-tubulin expression are shown. *n* = 3 mice per experimental group. (**G**) The cytosolic and mitochondrial proteins were obtained from pooled liver tissues from 5 to 6 mice per group. Representative bands of MFN 1/2, OPA1, COX IV, and α-tubulin expression are shown. *n* = 5–6 mice per experimental group. The relative band intensities were calculated using Image Lab software.

To assess the changes in mitochondrial function in the liver of ALS model mice, we first measured the amount of ATP in the liver of non-Tg and hSOD1^G93A^ Tg mice and found that it was increased by approximately 2.0-fold in Tg mice compared with that in non-Tg mice (Figure 7A). Interestingly, we found that the BJIGT and RZ combination significantly reduced ATP levels in the serum and liver of Tg mice compared to those in control mice by 0.1-fold and 0.3-fold, respectively (Figure 7B).

As mitochondrial OXPHOS is important for the maintenance of metabolism and survival, we investigated whether the mitochondrial OXPHOS system was impaired in the liver of ALS model mice. Using qPCR, we measured the relative gene expression levels of OXPHOS subunits (from *complex I* to *complex V*) in the livers of hSOD1^G93A^ mice. There was an overall increase compared with those in the non-Tg mice; the expression levels of *complex III*, *complex IV (1A),* and *complex V* were significantly increased in the liver, by 2.4-fold, 2.0-fold, and 2.6-fold, respectively (Figure 7C), indicating that mitochondrial OXPHOS processes are significantly impaired in the liver of hSOD1^G93A^ mice. Expression levels of OXPHOS subunits, especially *complex II*, *IV (1A)*, *IV (5A)*, and *V*, were significantly decreased in mice treated with combined BJIGT and RZ compared to those in control mice, by 0.8-fold, 0.6-fold, 0.4-fold, and 0.7-fold, respectively (Figure 7C). Administration of RZ alone also decreased the mRNA expression levels of *complex II* and *IV (5A)* by 0.8-fold and 0.5-fold, respectively, whereas it increased those of *complex I (ND1)* and *complex III* by 1.6-fold and 1.4-fold, respectively, compared with those in control mice. The mRNA expression level of *complex IV (1A)* was affected in mice treated with only BJIGT, by 0.6-fold. This result suggests that highly abnormal mitochondrial OXPHOS subunits in the liver of hSOD1^G93A^ mice were reduced by combining BJIGT and RZ. Although the activity of the *complex V* in the mitochondria of the liver was increased by 1.4-fold in Tg mice compared with that in non-Tg mice (Figure 7D). We found that the BJIGT and RZ combination significantly decreased the activity of *complex V* in the liver of Tg mice compared to that in control mice by 0.5-fold (Figure 7E).

To determine whether mitochondrial biogenesis was associated with increased ATP levels resulting in a dysregulated OXPHOS system, we examined the expression level of mitochondrial-fusion-related proteins, including MFN 1/2 and OPA1, in the liver of hSOD1^G93A^ mice. Mitochondria in the liver of Tg mice expressed higher levels of the mitochondrial proteins MFN 1/2 and OPA1 compared with that in non-Tg mice (Figure 7F). However, the expressions of MFN 1/2 and OPA1 were significantly reduced in liver mitochondria of mice in the BJIGT and RZ combination group compared to those in control mice, by 0.6- and 0.5-fold, respectively (Figure 7G). These results suggest that the BJIGT and RZ combination treatment may have an inhibitory effect on unnecessarily hyperfused mitochondria in the liver, thereby regulating ATP production and the OXPHOS system.

## 4. Discussion

Until now, RZ has been the most widely used ALS treatment, but it is limited in slowing the rate of disease, extending lifespan by 2–3 months only. Therefore, many attempts have been made to develop new treatments for ALS, including drug combination therapy, which is known to increase therapeutic effects and drug efficacy [22]. When herbs and drugs are co-administered, the pharmacological activity or toxicological effects may increase or decrease drug efficacy or side effects owing to interactions with herbs during drug metabolism.

The liver is one of the organs responsible for drug metabolism and herb–drug interactions [23]. Drug metabolism via the CYP system, which is involved in the metabolism of drugs and endogenous substrates, has emerged as an important factor in herb–drug interactions [24]. Therefore, hepatic CYP analysis is required to determine the suitability of new herb–drug combination therapies. CYP1A2 mainly metabolizes RZ via *N*-hydroxylation in the liver [25], and CYP3A4 is unlikely to be involved in RZ metabolism in humans. Although there are no reports on the metabolism of BJIGT in humans, previous studies have shown that BJIGT in CYP450 isozyme assays is unlikely to affect herb–drug interactions through modulation of human hepatic CYPs [26]. Furthermore, when drug combinations induce hepatic injury, the activities of several liver enzymes, including AST, ALT, ALP, and LDH, are dramatically elevated in the serum and liver [27,28]. Our data showed that BJIGT and RZ in combination reduced the activity of AST, ALT, ALP, and LDH in the serum and liver of hSOD1^G93A^ mice and showed no liver histopathological patterns. These results suggest that the combined administration of BJIGT and RZ does not adversely affect drug metabolism and liver function enzymes but rather effectively ameliorates functional problems in the livers of hSOD1^G93A^ mice.

Inflammation and cellular stress responses are closely associated with motor neuron death and ALS pathology [29]. A previous study has demonstrated that abnormal changes in the immune system owing to the elevation of peripheral NKT cell levels are associated with liver pathology and disease progression in hSOD1^G93A^ mice [11]. We also previously reported that the livers of hSOD1^G93A^ mice are associated with elevated inflammation, oxidative stress, and fibrosis-related protein expression following liver pathology and disease progression [13]. In this study, we found that the combined administration of BJIGT and RZ did not induce liver pathology, suggesting that this new therapeutic drug combination has the potential to effectively relieve inflammatory cytokines and cellular stress-related proteins in the liver of hSOD1^G93A^ mice.

ALS is a fatal, multifactorial disease, and increasing evidence implicates abnormalities in non-motor neurons and other organs, including the liver [2]. The liver is a pivotal metabolic organ that acts as a hub that connects various organs, including skeletal muscle and adipose tissue [30]. Growing evidence suggests that dysregulated energy metabolism in patients with ALS and mouse models is closely correlated with metabolic abnormalities in disease progression and susceptibility [31,32,33]. It has been reported that the activity of Mdh is increased in the synaptosomes and homogenates from the spinal cord and motor cortex in hSOD1^G93A^ mice in the early stages of the disease [34]. In addition, livers of hSOD1^G93A^ mice show significantly increased Mdh2 mRNA expression levels [2]. Consistent with those of previous reports, our results demonstrated that mRNA expression levels of Aco2, Mdh2, and Phkg1 in the liver of hSOD1^G93A^ mice were much higher than those in non-Tg mice. The expression levels of these metabolic-pathway-related factors were significantly reduced by the combined administration of BJIGT and RZ. It has been reported that hSOD1^G93A^ mice exhibit swollen and vacuolated mitochondria and disturbed mitochondrial distributions in motor neurons of the spinal cord [35]. Thus, mitochondria are considered an important potential target for understanding ALS progression and therapy. In the livers of hSOD1^G93A^ mice, ATP levels and gene expression levels of mitochondrial OXPHOS subunits were much higher than those in non-Tg mice, which were significantly attenuated by the combination of BJIGT and RZ. In particular, it is important to note that the expression of several complexes, including complex II, IV, and V, was significantly decreased in the livers of mice treated with both BJIGT and RZ. It is thought that the OXPHOS process is unnecessarily excessive, leading to an increase in ATP production. This is because metabolic alterations have been reported in other organs with impaired mitochondrial bioenergetics [2,36].

When cellular stress occurs, or energy is insufficient in an organ, mitochondria undergo elongation and hyperfusion, which is accompanied by increased ATP production to modulate metabolic stress owing to energy deprivation [37]. Dietary restriction is known to increase mitochondrial fusion by activating cyclic AMP levels and protein kinase A, resulting in increased cellular ATP levels [38]. This change suggests that conversion to mitochondrial fusion increases metabolism. Recent evidence suggests that the hyperfused state is permanent with an imbalance of mitochondrial fission–fusion under pathological conditions, leading to cytotoxic effects [39]. Based on these studies, the finding of abnormal mitochondrial fusion through increased protein expression of MFN1/2 in the liver suggests that impaired mitochondrial function is involved in the pathology of ALS. Thus, this study suggests that unnecessary OXPHOS is activated owing to abnormal mitochondrial fusion in the liver of hSOD1^G93A^ mice. Accordingly, excessive ATP is generated, and metabolism is disrupted. These detrimental events in the liver of hSOD1^G93A^ mice were modulated by the combined administration of BJIGT and RZ.

## 5. Conclusions

Collectively, we found that the combination of BJIGT and RZ did not affect drug metabolism, liver function, or hepatic histological patterns in the liver of hSOD1^G93A^ mice. The new combination investigated in this study effectively ameliorated inflammatory cytokines, cellular stress, and liver metabolic abnormalities and significantly suppressed metabolic pathway abnormalities and mitochondrial dysfunction in the liver of hSOD1^G93A^ mice. Therefore, this study shows that BJIGT and RZ are suitable for drug combination therapy, suggesting their potential as new treatments for ALS. Furthermore, further investigation is needed on the efficacy of BJIGT and RZ combination therapy on general symptoms and survival and to further explore the association of the liver, muscle, and spinal cord in the pathogenesis of ALS.

## Data Availability

All the data are available within the article or Supplementary Materials.

## Supplementary Materials

The following supporting information can be downloaded at: https://www.mdpi.com/article/10.3390/antiox11010173/s1, Table S1: Primers for quantitative reverse transcription PCR.
