# Supplementary material for: Combined Treatment with Herbal Medicine and Drug Ameliorates Inflammation and Metabolic Abnormalities in the Liver of an Amyotrophic Lateral Sclerosis Mouse Model
Source: Antioxidants (Basel). 2022 Jan 17;11(1):173. doi: 10.3390/antiox11010173 (PMC8773307; doi:10.3390/antiox11010173)
Supplement: Supplementary file 1 [file antioxidants-11-00173-s001.zip › antioxidants-1545573-supplementary.pdf]

Table S1. Primers for quantitative reverse transcription PCR.

Name	Primer and sequence (5'-3')
CYP1A2	Forward- CATCACAAGTGCCCTGTTCAAGC
	Reverse- AATGCTCCAGGTGATGGCTGTG
CYP3A4	Forward- CTGAGCTTTCTCAGTGTCTGTG
	Reverse- GATCCCATGAGAAACGGTGAAG
IL-1β	Forward- TCTATACCTGTCCTGTGTAATG
	Reverse- GCTTGTGCTCTGCTTGTG
TNF-α	Forward- GTGGAACTGGCAGAAGAG
	Reverse- AATGAGAAGAGGCTGAGAC
IL-6	Forward- CCCCAATTTCCAATGCTCTCC
	Reverse- CGCACTAGGTTTGCCGAGTA
Aco1	Forward- CCATCCGTGATGTTAGGAGCAG
	Reverse- GACAGGTAAGGCATGACTCCAC
Aco2	Forward- ACTGTGGCAGAGAAGGAAGGCT
	Reverse- CAATGCCTGCTTAGCCACAGCT
Mdh1	Forward- TTCTGGACGGTGTCCTGATGGA
	Reverse- TAGGACAGCCACATCCAGGTCT
Mdh2	Forward- TCACTCCTGCTGAAGAACAGCC
	Reverse-CCTTTGAGGCAATCTGGCAACTG
Pygl	Forward- GGCAGAAGTGGTGAACAATGACC
	Reverse- TCCGATAGGTCTGTGGCTGGAA
Phkg1	Forward- GCTCACAGACTTCGGGTTTTCC

	Reverse- ATGTCCACCTCCTTCCCATAGC
Phkg2	Forward- ACCCAGGCTATGGCAAGGAAGT
	Reverse- TGCGTAGCATCAGGATTTGGCG
Gbe1	Forward- CTCTGTTCTCCAACTTCCCAGG
	Reverse- CAAGGTAGCGTCGATTGGTGAG
Complex I (ND1)	Forward- CAGGATGAGCCTCAAACTCC
	Reverse- GGTCAGGCTGGCAGAAGTAA
Complex I (ND2)	Forward- AGGGATCCCACTGCACATAG
	Reverse- CCTATGTGGGCAATTGATGA
Complex II	Forward- ACAAATGGTCTCTTCCTATGGCA
	Reverse- CCCCTCCACTCAAGGCTATTC
Complex III	Forward- ACGTCCTTCCATGAGGACAA
	Reverse- GAGGTGAACGATTGCTAGGG
Complex IV (1A)	Forward- CTTTTATCCTCCCAGGATTTGG
	Reverse- GCTAAATACTTTGACACCGG
Complex IV (5A)	Forward- CTTTAAATGAATTGGGAATCTCCAC
	Reverse- GCCCATCGAAGGGAGTTTACA
Complex V	Forward- AATTACAGGCTTCCGACACAAAC
	Reverse- TGGAATTAGTGAAATTGGAGTTCCT
GAPDH	Forward- CCTCGTCCCGTAGACAAA
	Reverse- AATGAAGGGGTCGTTGATG

Abbreviations: CYP1A2, Cytochrome P450 1A2; CYP3A4, Cytochrome P450 3A4; IL-1β,

Interleukin-1β; TNF-α, Tumor necrosis factor-α; IL-6, Interleukin-6; Aco1, Aconitase 1; Aco2, Aconitase 2; Mdh1, Malate dehydrogenase 1; Mdh2, Malate dehydrogenase 2; Pygl, glycogen phosphorylase; Phkg1, Phosphorylase kinase catalytic subunit gamma 1; Phkg2, Phosphorylase kinase catalytic subunit gamma 2; Gbe1, glycogen branching enzyme 1; GAPDH, Glyceraldehyde 3-phosphate dehydrogenase
